# Novel induction of broad-spectrum antibiotics by the human pathogen *Legionella*

**DOI:** 10.1128/msphere.00120-24

**Published:** 2024-06-18

**Authors:** Carson J. Shin, Tamara J. O'Connor

**Affiliations:** 1 Department of Biological Chemistry, The Johns Hopkins University School of Medicine, Baltimore, Maryland, USA; University of Nebraska Medical Center College of Medicine, Omaha, Nebraska, USA

**Keywords:** honey, *Legionella*, *Bacillus*, antibiotic, bacterial pathogen

## Abstract

The majority of antibiotics are natural products, with microorganism-generated molecules and their derivatives being the most prevalent source of drugs to treat infections. Thus, identifying natural products remains the most valuable resource for novel therapeutics. Here, we report the discovery of a series of dormant bacteria in honey that have bactericidal activity toward *Legionella*, a bacterial pathogen that causes respiratory disease in humans. We show that, in response to bacterial products secreted by *Legionella*, the honey bacteria release diffusible antimicrobial molecules. Remarkably, the honey bacteria only produce these molecules in response to *Legionella* spp., when compared to a panel of 24 bacterial pathogens from different genera. However, the molecules induced by *Legionella* have broad activity against several clinically important pathogens, including many high-priority pathogens. Thus, *Legionella* spp. are potent drivers of antimicrobial molecule production by uncharacterized bacteria isolated from honey, providing access to new antimicrobial products and an unprecedented strategy for discovering novel antibiotics.

**IMPORTANCE:**

Natural products generated by microorganisms remain the most viable and abundant source of new antibiotics. However, their discovery depends on the ability to isolate and culture the producing organisms and to identify conditions that promote antibiotic production. Here, we identify a series of previously undescribed bacteria isolated from raw honey and specific culture conditions that induce the production of antimicrobial molecules that are active against a wide variety of pathogenic bacteria.

## INTRODUCTION

There is an urgent need for new antibiotics to address the growing threat of antimicrobial resistance (1). Natural products are the basis of most approved antibiotics and remain the most valuable resource for the discovery of novel therapeutics (2). Antibiotic-producing microorganisms are highly diverse, often requiring specific culture conditions for their identification and propagation. Moreover, the production of antimicrobial molecules is often associated with a fitness cost, and thus, their biosynthesis is highly regulated (3, 4). As a consequence, their discovery greatly depends on culture and drug production conditions. These specialized conditions are key to identifying new bacteria and their bioactive natural products.

Honey has been used as a traditional medicine for centuries to treat a variety of ailments, including bacterial infections (5–17). The antimicrobial properties of honey are attributed to the high concentrations of glucose and fructose, which impart its hygroscopic properties (18, 19), enzymes such as glucose oxidase that converts glucose to gluconic acid and hydrogen peroxide, causing its low pH and high levels of reactive oxygen species (ROS) (20), and a number of antimicrobial peptides, including defensin-1 and the jellein family peptides (21, 22). However, for some honey variants, there are additional, as yet undefined features that contribute to their potency (23–25). The composition of honey depends on several factors but is mainly determined by the species of bees and the local plant flora of the beehive. As a consequence, the antimicrobial properties of individual honey types vary considerably, as does their potency toward different bacteria, such as *Bacillus*, *Escherichia*, *Salmonella*, *Pseudomonas*, *Listeria*, and *Staphylococcus* (11–17, 26–28).

Honey itself is generally considered to be highly sterile, as most microorganisms are unable to survive the highly potent antimicrobial environment. However, a number of spore-forming microorganisms have been isolated from honey, including species of the bacterial genera *Bacillus*, *Paenibacillus*, *Lysinibacillus*, and *Clostridium*, and several fungal genera including *Aspergillus* and *Candida* (29–36). The dormant, highly resilient spore form allows these microorganisms to withstand the harsh environment of honey. The primary sources of bacterial contamination include pollen, nectar, flowers, soil, and the digestive tracts of bees (28, 37–40). Thus, the microbiomes of individual honey variants are both region specific and highly diverse.

*Legionella pneumophila* is a Gram-negative bacterial pathogen that causes pneumonia in humans known as Legionnaires’ disease (41). *L. pneumophila* are ubiquitous in nature in water and soil (42, 43) and are environmentally acquired, with human exposure occurring through the inhalation of contaminated water aerosols (44). Here, we examined the antimicrobial activity of 11 types of raw honey from a range of geographical locations against *L. pneumophila*. While *L. pneumophila* was largely resistant to the antimicrobial effects of honey itself, uncharacterized bacteria isolated from the honey showed potent antimicrobial activity toward *L. pneumophila* and a diverse panel of other clinically relevant *Legionella* spp. Notably, antimicrobial molecule production by the honey bacteria was only induced in response to *Legionella* spp. when compared to a panel of other bacterial pathogens, defining distinct conditions required for molecule production. However, the molecules produced showed broad specificity toward other human pathogens, including several high-priority pathogens (1). Thus, bacteria isolated from honey represent an untapped resource of potentially broad-spectrum antibiotics, with *Legionella* spp. functioning as specific inducers of antibiotic production.

## RESULTS

### *L. pneumophila* is largely resistant to the canonical antimicrobial properties of honey

The impact of honey and its antimicrobial properties on a number of human pathogens has been examined in detail (11–17). However, one pathogen that has not been evaluated in this regard is *L. pneumophila*. Since the composition, and thus antimicrobial properties of individual honey types vary, we systematically compared the antimicrobial effects of 11 types of honey from various regions across the globe (Fig. S1; Table S1). To do this, raw honey was diluted 50% (vol/vol) in sterile water and disk diffusion assays (45) were performed, in which honey samples were spotted on filter disks placed on a lawn of *L. pneumophila* and the impact of the honey on *L. pneumophila* growth was measured (Fig. S2A through C). Of the 11 honey variants tested, Manuka honey was the most potent, giving rise to a modest zone diameter of inhibition (ZDI) of 11 ± 1 mm compared to the untreated control, with a reported ZDI of 6 mm, the diameter of the filter disk itself (Fig. S2B). African and Tualang honey also inhibited *L. pneumophila* growth but to a lesser extent (Fig. S2B). In contrast, none of the other honey types had any impact on *L. pneumophila* growth (Fig. S2B and C). This was not due to lack of activity, as all 11 honey variants were able to restrict the growth of *Escherichia coli* to some extent (Fig. S2D through F). While the antimicrobial activities of the honey variants against *E. coli* could be attributed to typical features (low pH, ROS, and antimicrobial peptides) (Fig. S2G and H), this was not the case for *L. pneumophila*. Instead, while ROS played a minor role in impairing *L. pneumophila* growth, the inability of altering the pH, inactivating ROS, or removing peptides to abolish the antimicrobial activity of honey toward *L. pneumophila* (Fig. S2I) indicated there were other unidentified contributing factors.

### Bacteria isolated from raw honey have antimicrobial activity against *L. pneumophila*

**Fig 1:**
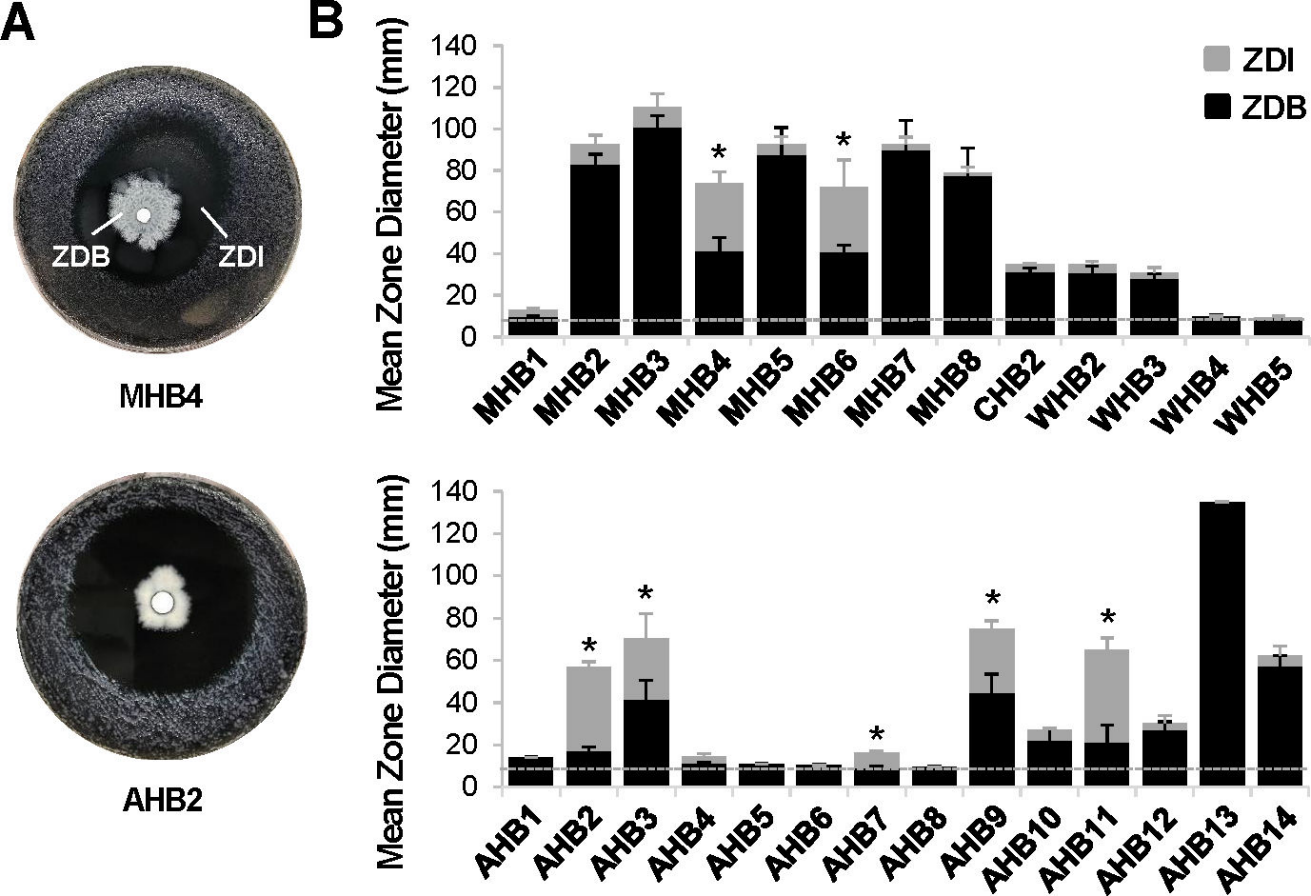
Bacteria isolated from honey exhibit antibacterial activity against *L. pneumophila*. (**A**) Bacteria isolated from honey inhibit *L. pneumophila* growth. Disk diffusion assays spotting honey bacteria on filter discs placed on a lawn of *L. pneumophila*. The zone diameter of honey bacteria outgrowth (ZDB) from filter disks and the zone diameter of inhibition of *L. pneumophila* growth (ZDI) are indicated. (**B**) Disk diffusion assay ZDBs for the indicated honey bacteria and the associated ZDIs of *L. pneumophila* growth as in panel** A** were quantified. MHB, CHB, WHB, and AHB are Manuka, clover, wildflower, and African honey bacteria, respectively. A dotted line indicates the diameter of the filter disk (6 mm) and thus represents no honey bacteria growth or zone of inhibition of *L. pneumophila* growth. Data are the mean of three to six biological replicates, each consisting of one to two technical replicates each. Error bars indicate ±standard deviation. An asterisk indicates a two-tailed Student’s *t*-test *P* value of <0.05 comparing the ZDI to the ZDB.

In the course of performing initial disk diffusion assays using unfiltered honey samples, a number of non-*Legionella* bacteria were found growing out from the filter disks (Fig. S3) (subsequently referred to as honey bacteria) with varying colony morphologies and pigmentations (Fig. S4). These included 14 African honey bacteria (AHB), 2 clover honey bacteria, 5 wildflower honey bacteria, and 11 Manuka honey bacteria (MHB). 16S sequencing identified the majority of these bacteria as members of the spore-forming genera *Bacillus*, *Paenibacillus*, *Lysinobacillus*, *Rummeliibacillus*, or *Niallia*, with two exceptions, AHB1 and MHB7, which were identified as *Staphylococcus epidermidis* and *Staphylococcus capitis*, respectively (Table S2). Notably, for a number of the honey bacteria, distinct zones of inhibition of *L. pneumophila* growth surrounding the bacterial colonies were observed (Fig. S3). To examine this in detail, individual bacteria were colony purified and then used to perform disk diffusion assays. Honey bacteria were spotted on filter disks placed on a lawn of *L. pneumophila*, and the zone diameter of honey bacteria (ZDB) outgrowth from the filter disk and the ZDI of *L. pneumophila* growth (Fig. 1A) were measured. Of the 30 honey bacteria tested, 2 from Manuka honey (MHB4 and MHB6) and 5 from African honey (AHB2, AHB3, AHB7, AHB9, and AHB11) were able to prevent *L. pneumophila* growth, generating ZDIs that extended from 7 ± 1 mm to 43 ± 6 mm beyond the boundary of the honey bacteria outgrowth (Fig. 1B). These results demonstrated that multiple bacterial species present in honey produce antimicrobial molecules with modest to highly potent activity against *L. pneumophila*.

**Fig 2:**
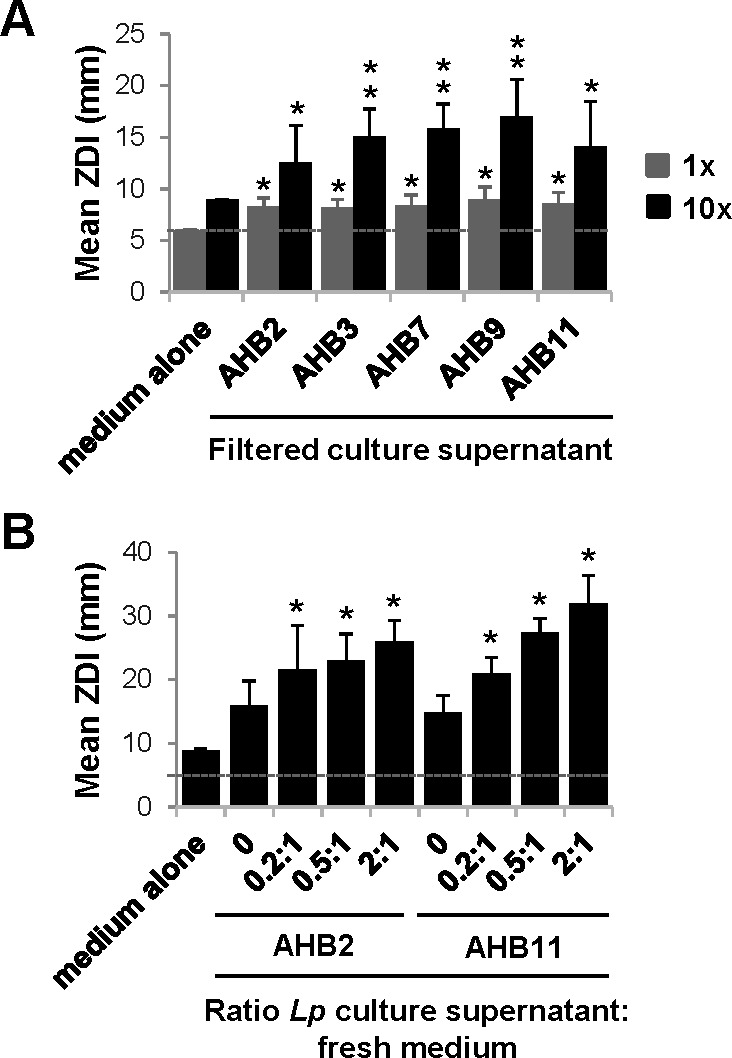
*L. pneumophila* induces the production of a secreted, diffusible antimicrobial molecule by honey bacteria. (**A**) Honey bacteria secrete antimicrobial molecules that inhibit *L. pneumophila* growth. Culture supernatants of the indicated honey bacteria were harvested, filter sterilized, and used directly (1×) or concentrated 10-fold (10×). Samples were examined for their ability to impair *L. pneumophila* growth in disk diffusion assays, comparing the zone diameter of inhibition (ZDI) to medium alone. (**B**) Honey bacteria produce antimicrobial molecules in response to exposure to *L. pneumophila. L. pneumophila* culture supernatants were harvested and filter sterilized. Honey bacteria were then cultured in the presence or absence of *L. pneumophila* filtered culture supernatants. Honey bacteria culture supernatants were then harvest, concentrated 10×, and used in disk diffusion assays as in panel **A **(see Materials and Methods and Fig. S5). (**A and B**) A dotted line indicates the diameter of the filter disk (6 mm) and thus represents no inhibition. Data are the mean of three to nine biological replicates. Error bars indicate ±standard deviation. Asterisks indicate two-tailed Student’s *t*-test *P* values: *P < 0.02, **P < 0.005 compared to medium alone.

The ability of the honey bacteria to impair *L. pneumophila* growth across a large distance suggested the effects were due to a secreted, diffusible molecule. To test this, the culture supernatants of the five most potent African honey bacteria (AHB2, AHB3, AHB7, AHB9, and AHB11) were filter sterilized and evaluated in disk diffusion assays for their ability to impair *L. pneumophila* growth. For AHB7, similar growth inhibition was observed between actively growing honey bacteria on solid medium and filtered culture supernatants, with ZDIs of 7 ± 1 mm and 8 ± 1 mm, respectively (Fig. 1B and 2A). In contrast, AHB2, AHB3, AHB9, and AHB11 filtered culture supernatants showed significantly smaller effects on *L. pneumophila* growth, with ZDIs averaging 9 ± 1 mm (Fig. 2A) compared to ZDIs of 23–43 mm in disk diffusion assays with actively growing bacteria (Fig. 1B). One possible explanation for this discrepancy was that the relative concentration of the active molecules in liquid culture was lower than that in solid medium. To test this hypothesis, honey bacteria filtered culture supernatants were concentrated 10-fold and disk diffusion assays were repeated. Under these conditions, inhibition of *L. pneumophila* growth was enhanced for all five honey bacteria filtered culture supernatants (Fig. 2A). This was not due to the potentially toxic effects of higher levels of medium constituents such as iron after concentration, as 10-fold concentrated medium alone did not produce a similar effect (Fig. 2A). In the case of AHB7, the ZDI of concentrated, filtered culture supernatant (16 ± 2 mm) (Fig. 2A) was greater than that of actively growing honey bacteria (7 ± 1 mm) (Fig. 1B). In contrast, while the ZDIs for AHB2 (13 ± 4 mm), AHB3 (15 ± 3 mm), AHB9 (17 ± 4 mm), and AHB11 (14 ± 4 mm) filtered culture supernatants increased after concentration (Fig. 2A), the resulting ZDIs were still smaller than those produced by bacteria growing on solid medium (40 ± 2 mm, 29 ± 12 mm, 30 ± 4 mm and 43 ± 6 mm, respectively) (Fig. 1B). The greatest discrepancy was observed for AHB2 and AHB11, which were notably the most potent bacteria on solid media. These results validated the role for a secreted molecule(s) produced by honey bacteria to inhibit *L. pneumophila* growth. In addition, they indicated that for the majority of honey bacteria tested, when grown in liquid culture, production of the active molecule(s) was less efficient than on solid medium.

### *L. pneumophila* induces antimicrobial molecule production by honey bacteria

One major difference between honey bacteria growing on solid medium in disk diffusion assays and honey bacteria growing in isolation in liquid culture was the presence of *L. pneumophila*. Thus, it was possible that the low level of active molecule production in liquid was due to the absence of *L. pneumophila*. To determine whether *L. pneumophila* induced antimicrobial molecule production by the honey bacteria, *L. pneumophila* culture supernatants were harvested, filter sterilized, and then added to cultures of honey bacteria (see Materials and Methods and Fig. S5 for details), focusing on AHB2 and AHB11 that had the greatest potency on solid medium (Fig. 1B) but the weakest effect when their culture supernatants were examined (Fig. 2A). In both cases, adding *L. pneumophila* filtered culture supernatant to honey bacteria cultures resulted in a dose-dependent increase in the ZDI in disk diffusion assays (Fig. 2B), with a ratio of *L. pneumophila* filtered culture supernatant to fresh medium of 2:1 increasing the ZDIs of AHB2 from 8 ± 1 mm to 26 ± 3 mm and AHB11 from 9 ± 1 mm to 32 ± 4 mm, 3.2-fold and 3.5-fold, respectively. Collectively, these results demonstrated that *L. pneumophila* induces antimicrobial molecule production by AHB2 and AHB11, and this is mediated by a diffusible molecule(s) that *L. pneumophila* secretes or releases into the extracellular milieu.

### Honey bacteria antibacterial molecules are bactericidal toward *L. pneumophila*

**Fig 3:**
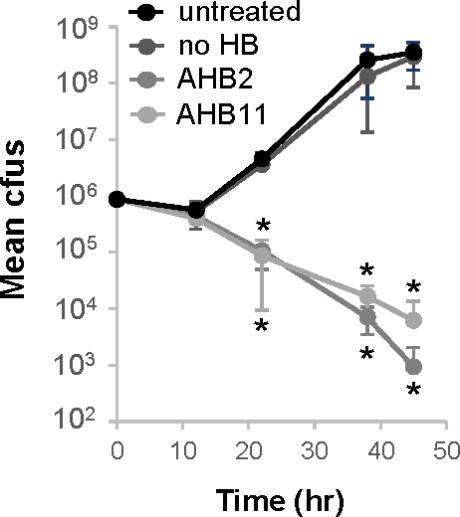
Honey bacteria antimicrobial molecules are bacteriostatic toward *L. pneumophila*. Concentrated filtered culture supernatants of honey bacteria exposed to *L. pneumophila*, as described in Fig. 2B and illustrated in Fig. S5, were added to *L. pneumophila* liquid cultures, and *L. pneumophila* numbers were monitored over time based on colony-forming units on solid medium. Data are the mean of four biological replicates. Error bars indicate ±standard deviation. An asterisk indicates a two-tailed Student’s *t*-test *P* value of <0.05 compared to untreated, *L. pneumophila* growth in fresh bacteriological medium. HB, honey bacteria.

Antimicrobial molecules can impede bacterial replication, exhibiting bacteriostatic activity or can actively promote bacterial cell death, as in the case of bactericidal agents. To determine whether the AHB2- and AHB11-produced molecules were bacteriostatic or bactericidal, their production by AHB2 and AHB11 was induced with filtered *L. pneumophila* culture supernatant, and honey bacteria culture supernatants were harvested, filter sterilized, and concentrated 10-fold (as in Fig. 2) (see Materials and methods and Fig. S5 for details). *L. pneumophila* was then cultured in liquid medium in the presence or absence of AHB2 or AHB11 concentrated, filtered culture supernatant and at regular intervals, viable *L. pneumophila* was quantified by plating aliquots on solid medium and enumerating colony-forming units (CFUs). In the absence of honey bacteria culture supernatant, *L. pneumophila* grew greater than 100-fold in a 48-h period (Fig. 3). In contrast, addition of AHB11 culture supernatant resulted in a greater than 2-log reduction in CFUs relative to the starting inoculum and a 100-fold decrease in viable bacteria over the 48-h experiment (Fig. 3). Even more striking, addition of AHB2 culture supernatant resulted in a greater than 3-log reduction in CFUs and a 1,000-fold decrease in viable bacteria (Fig. 3). The decrease in viability was not due to the presence of concentrated medium constituents as *L. pneumophila* grew at the same rate in concentrated bacteriological medium containing filtered *L. pneumophila* culture supernatant but lacking honey bacteria as in fresh medium (Fig. 3). Based on the Clinical and Laboratory Standards Institute (CLSI) definition of bactericidal compound activity (46), the >4 log difference in bacteria counts in the presence and absence of honey bacteria supernatant demonstrated that AHB2 and AHB11 produce and secrete an antimicrobial molecule that provides sustained bacterial cell killing of *L. pneumophila*.

### Genome sequencing of AHB2 and AHB11 reveals distinct differences between them

16S sequencing of AHB2 and AHB11 indicated they are both members of the *Bacillus* genus (Table S2). Subsequent whole genome sequencing of AHB2 (47) and AHB11 (48) further defined these bacteria as *Bacillus safensis*, based on multi-locus sequencing typing of 16S, 23S, *rpoB*, *gyrB*, and *recA* (Table S2). However, with only 90% gene conservation between them and a set of 732 genes unique to one or the other, they represent two distinct strains.

Members of the *Bacillus* genus are known producers of a variety of antimicrobial molecules (49). However, *Bacillus safensis* spp., in particular, are known for their production of antifungal (50–52) rather than antibacterial molecules. A cursory examination of the *B. safensis* AHB2 and AHB11 genomes using the antibiotics and secondary metabolite analysis shell(AntiSMASH) (53) predicts the presence of bacteriocins, including ribosomally synthesized and post-translationally modified peptides (RiPPs), non-ribosome peptides (NRPs), polyketides, terpenes, siderophores, and β-lactone biosynthetic genes, providing a number of potential sources of their antimicrobial activities (Table S3). Notably, with the exception of a homolog of the plantazolicin gene (encoding an ultra-narrow spectrum RiPP [54]) in AHB11, to our knowledge, these genes have not been characterized to date, suggesting they are likely to catalyze the biosynthesis of unique molecules. However, there may be other, as yet, unidentified molecules encoded within their genomes that are responsible for the antimicrobial activities induced in response to *L. pneumophila*.

### AHB2 and AHB11 active molecules are small, protease resistant, and thermostable

**Fig 4:**
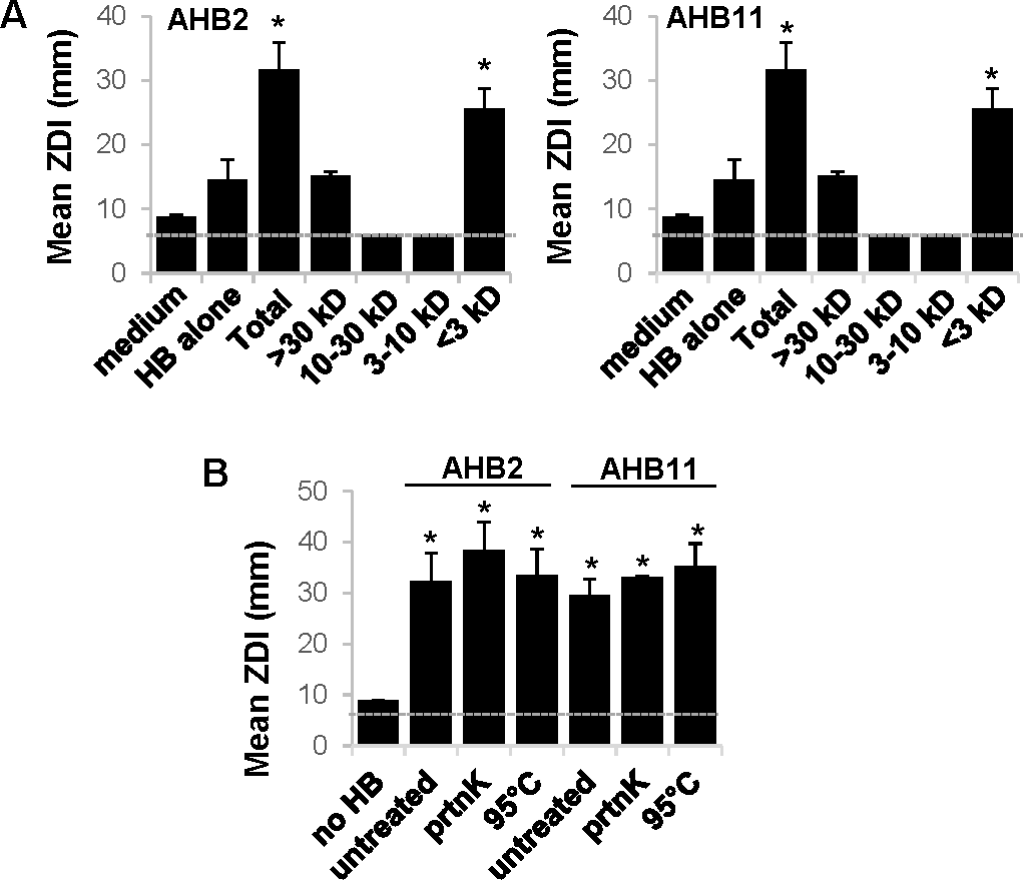
AHB2 and AHB11 active molecules are small, protease resistant and thermostable. (**A**) AHB2 and AHB11 active molecules are less than 3 kDa. Disk diffusion assays measuring growth inhibition of *L. pneumophila* by concentrated, filtered culture supernatants of AHB2 (left panel) or AHB11 (right panel) after exposure to *L. pneumophila* supernatant (total) and sequentially fractionated by filter centrifugation using the indicated molecular weight cutoffs. HB alone indicates concentrated, filtered culture supernatants of AHB2 or AHB11 not exposed to *L. pneumophila* supernatant. (**B**) AHB2 and AHB11 active molecules are resistant to proteolysis and thermostable. Disk diffusion assays as in which filtered culture supernatants of AHB2 or AHB11 were treated with proteinase K (prtnK) at 37°C for 45 min or heated to 95°C for 10 min. (**A and B**) Data are the mean ± standard deviation of three to four biological replicates, each consisting of two technical replicates. *P < 0.05 comparing the ZDI to medium alone (**A**) or no HB (**B**). A dotted line indicates the diameter of the filter disk (6 mm). HB, honey bacteria.

To further elucidate the nature of the AHB2 and AHB11 active molecules, we first examined the size of the molecules. To do this, AHB2 or AHB11 were exposed to *L. pneumophila* supernatant, and then the honey bacteria culture supernatants were harvested, filtered and subjected to sequential size fractionation using successively smaller exclusion limit filters. Each fraction was then concentrated and used in disk diffusion assays to test for inhibition of *L. pneumophila* growth. For both AHB2 and AHB11 honey bacteria, the <3-kDa fractions exhibited antimicrobial activity toward *L. pneumophila* that was comparable to the total, unfractionated sample (Fig. 4A). Conversely, none of the other fractions (3–10 kDa, 10–30 kDa, and >30 kDa) showed activity above concentrated filtered supernatant of honey bacteria that were not exposed to *L. pneumophila* supernatant (Fig. 4A). Next, we tested the susceptibility of the molecules to proteolytic digestion and heat by treating samples with proteinase K or high temperature and then performing disk diffusion assays. Neither treatment reduced the potency of the AHB2 or AHB11-generated molecules with the corresponding ZDIs remaining similar to that of the untreated samples (Fig. 4B). Thus, the AHB2 and AHB11 active molecules are relatively small, resistant to protease treatment, and thermostable, indicating they are unlikely to be non-post-translationally modified peptides or large proteins.

### Honey bacteria have broad-spectrum activity against multiple *Legionella* species

**Fig 5:**
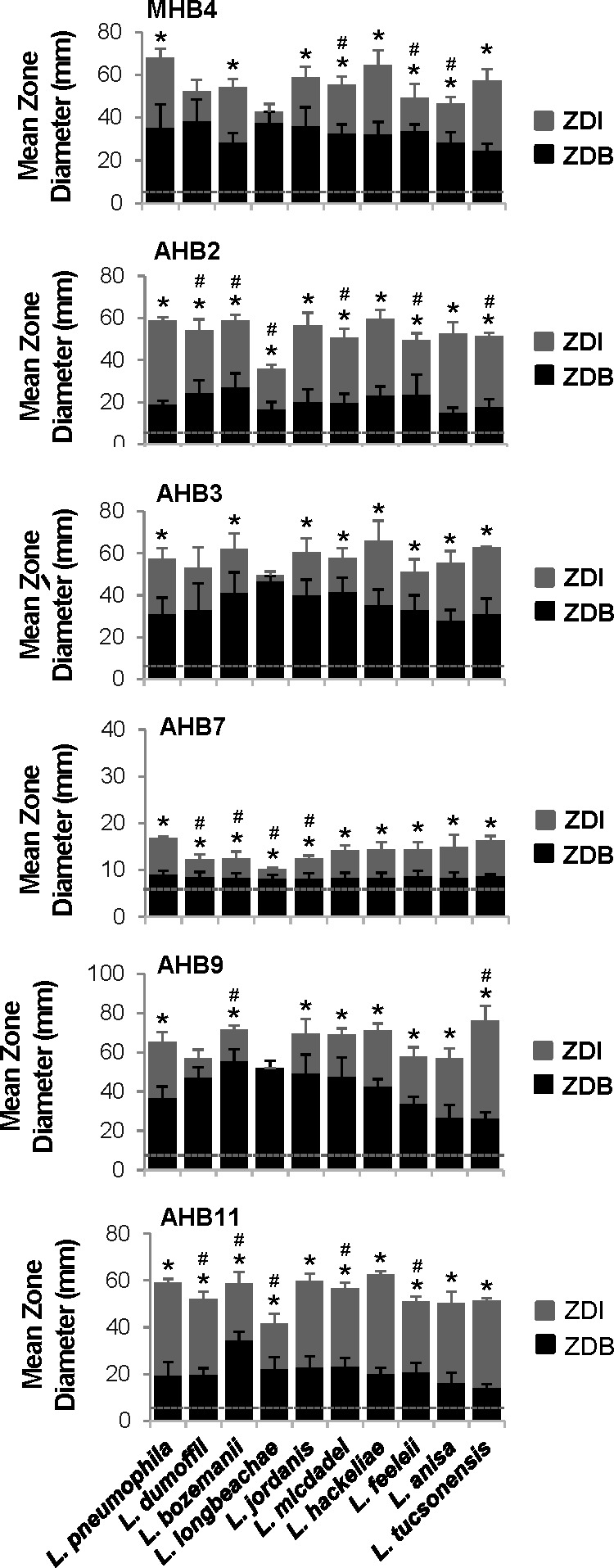
Bacteria isolated from honey exhibit broad specificity against members of the *Legionella* genus. Disk diffusion assays measuring growth inhibition of the indicated *Legionella* species by the indicated honey bacteria isolates, based on comparing the zone diameter of honey bacteria (ZDB) outgrowth to the zone diameter of inhibition (ZDI) of *L. pneumophila* growth as in Fig. 1. A dotted line indicates the diameter of the filter disk (6 mm). Data are the mean of three to five biological replicates. Error bars indicate ±standard deviation. An asterisk indicates a Student’s *t*-test *P* value of <0.05 comparing the ZDI to the ZDB. A number sign indicates a two-tailed Student’s *t*-test *P* value of <0.05 comparing the ZDI of the indicated species to that of *L. pneumophila*. HB, honey bacteria.

The *Legionella* genus encompasses 68 species (www.bacterio.net/legionella.html). While *L. pneumophila* is responsible for the majority of clinical cases, several other species have been reported to frequently cause disease, with *Legionella longbeachae*, *Legionella bozemanii*, *Legionella micdadei*, *Legionella feeleii*, *Legionella dumoffii*, *Legionella anisa*, *Legionella hackeliae*, *Legionella jordanis*, and *Legionella tucsonensis* being among the most prevalent (55, 56). To compare the effects of the more potent honey bacteria against different species of *Legionella* commonly associated with disease, the ability of MHB4, AHB2, AHB3, AHB7, AHB9, and AHB11 to inhibit their growth was compare to *L. pneumophila* by disk diffusion assay. For MHB4, AHB3, and AHB9, differential susceptibility was observed across the nine *Legionella* spp. examined (Fig. 5). For example, AHB9 was less potent toward *L. bozemanii* (16 ± 2 mm), similarly potent toward *L. hackeliae* (28 ± 3 mm), and more potent toward *L. tucsonensis* (50 ± 8 mm) when compared to *L. pneumophila* (28 ± 5 mm), whereas MHB4 was similarly potent toward all three of these species (26 ± 4 mm, 32 ± 7 mm, 33 ± 5 mm, and 22 ± 4 mm, respectively). Most notably, two species, *L. dumoffii* and *L. longbeachae*, were resistant to the effects of MHB4, AHB3, and AHB9 (Fig. 5). However, this was not due to lack of growth of the honey bacteria in the presence of *L. dumoffii* and *L. longbeachae*, as similar ZDBs were observed compared to those in the presence of susceptible *Legionella* spp. Instead, this could be because these honey bacteria respond to different inducers that are not produced by these *Legionella* spp., differences in the activities of the molecules produced by these honey bacteria, or resistance mechanisms inherent to *L. dumoffii* and *L. longbeachae*. In contrast, AHB2, AHB7, and AHB11 exhibited antimicrobial activity toward all nine *Legionella* spp. tested (Fig. 5), demonstrating these three honey bacteria produce antimicrobial molecules that have broader specificity against members of this genus, with AHB2 and AHB11 also exhibiting high potency toward multiple clinically important *Legionella* spp.

### The antimicrobial response of honey bacteria is specific to the *Legionella* genus

**Fig 6:**
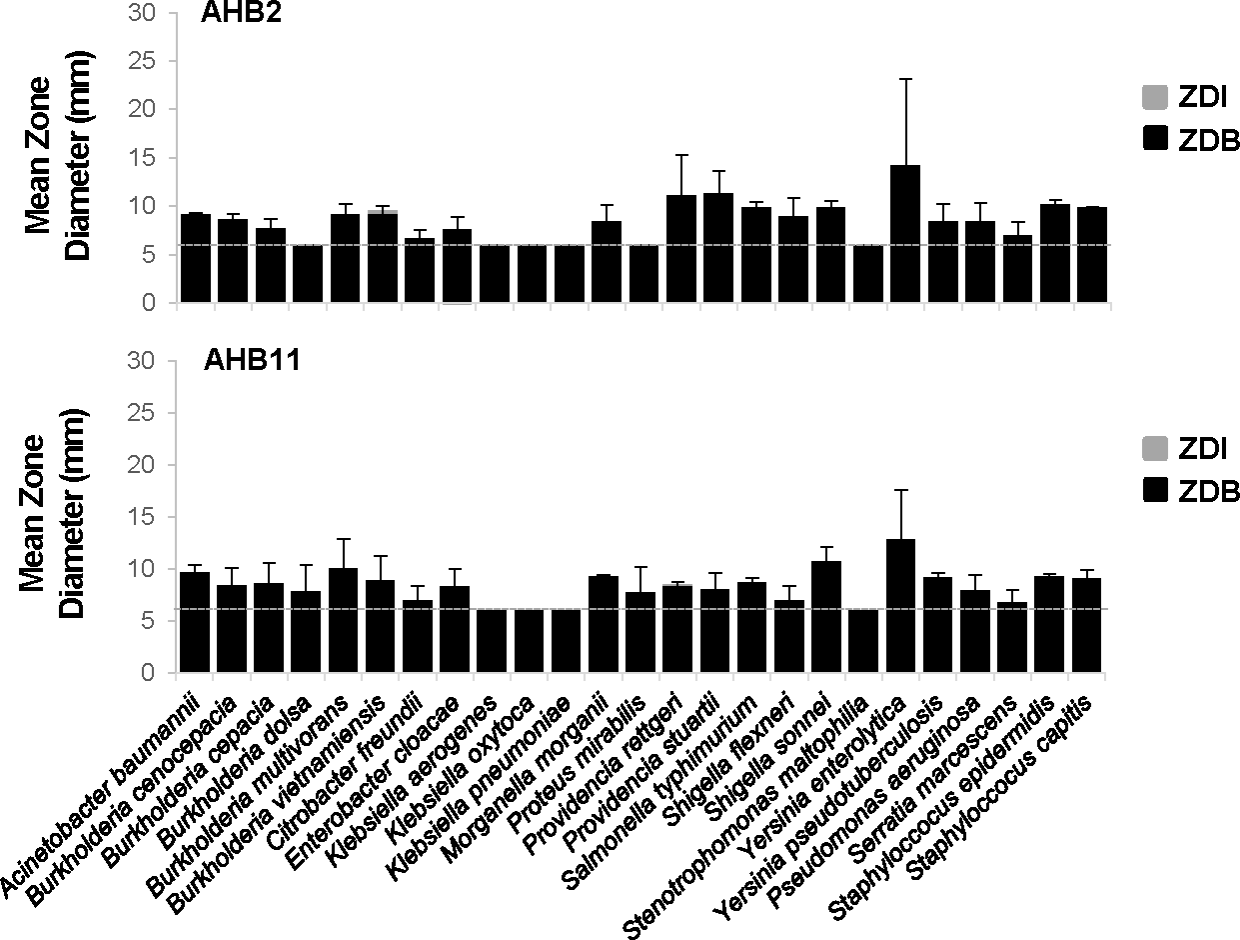
The antimicrobial response of honey bacteria is specific to the *Legionella* genus. Disk diffusion assays measuring growth inhibition of the indicated pathogenic bacteria by AHB2 (top panel) and AHB11 (bottom panel) honey bacteria, based on the zone diameter of inhibition (ZDI) of the pathogenic bacteria compared to the zone diameter of honey bacteria (ZDB) outgrowth, as in Fig. 1. A dotted line indicates the diameter of the filter disk (6 mm). Data are the mean of three to four biological replicates. Error bars indicate ±standard deviation. An asterisk indicates a two-tailed Student’s *t*-test *P* value of <0.05 comparing the ZDI to the ZDB.

Since AHB2 and AHB11 showed broad specificity against multiple *Legionella* species and were the most potent against *Legionella*, we examined their activity toward an assorted panel of human bacterial pathogens, including respiratory, enteric, skin, and urinary tract pathogens (Table S4). Intriguingly, growth of all 25 pathogens tested was not affected by either honey bacterium using disk diffusion assays in which bacteria were plated directly on filter disks, with no ZDIs detected (Fig. 6). In the case of *Klebsiella* species, *Proteus mirabilis*, and *Stenotrophomonas maltophilia*, this may have been due to lack of viability and/or growth of the honey bacteria in the presence of these organisms, as no ZDB was detected for either AHB2 and AHB11. These results suggested that the antimicrobial activity of AHB2 and AHB11 are highly specific to the *Legionella* genus.

### The antimicrobial molecules produced by honey bacteria in response to *Legionella* restrict the growth of other bacterial pathogens

**Fig 7:**
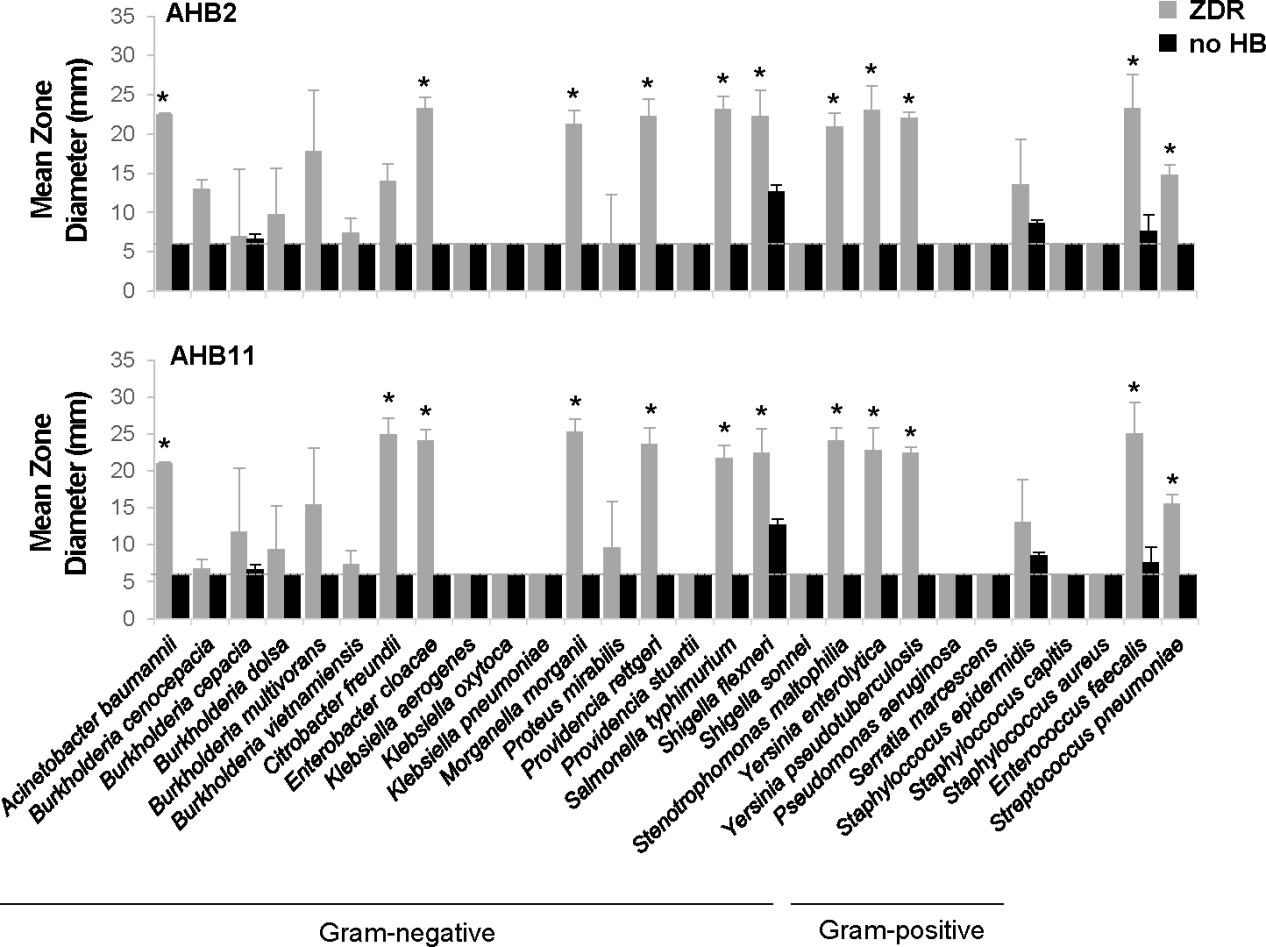
Antimicrobial molecules produced in response to *Legionella* have broad specificity against other bacterial pathogens. Disk diffusion assays measuring growth restriction of the indicated pathogenic bacteria by concentrated, filtered culture supernatants of AHB2 and AHB11 honey bacteria after exposure to *L. pneumophila* filtered culture supernatants (as in Fig. 2B). The zone diameter of pathogenic bacteria restriction (ZDR) was compared to the zone diameter of similar samples lacking honey bacteria (no HB). A dotted line indicates the diameter of the filter disk (6 mm). Data are the mean of three to four biological replicates for Gram-negative bacteria and four to six biological replicates for Gram-positive bacteria. Error bars indicate ±standard deviation. An asterisk indicates a two-tailed Student’s *t*-test *P* value of <0.05 comparing the ZDI in the presence of honey bacteria to *L. pneumophila* filtered culture supernatant lacking honey bacteria (no HB). Disk diffusion quality control assays using CSLI-recommended *E. coli* ATCC25922 and *S. aureus* ATCC25923 and gentamicin disk content were in the quality control range.

The lack of growth inhibition observed for non-*Legionella* bacterial pathogens exposed to AHB2 or AHB11 could be due to their lack of susceptibility to the molecule(s) generated by the honey bacteria, the existence of resistance mechanisms, or their inability to induce production of the molecule(s). To test the latter, we induced antimicrobial molecule production by honey bacteria by exposing them to *L. pneumophila* supernatants and then tested whether their concentrated, filtered culture supernatants were able to impact growth of the same panel of pathogens. While neither AHB2 or AHB11 supernatants resulted in growth inhibition, for nine genera of Gram-negative pathogens examined (*Acinetobacter*, *Citrobacter*, *Enterobacter*, *Morganella*, *Providencia*, *Salmonella*, *Shigella*, *Strenotrophomonas*, *Yersinia*, and, to a lesser extent, *Burkholderia*), we observed distinct zone diameters of growth restriction (ZDRs) (Fig. 7), defined by visibly less dense bacterial growth surrounding the filter disk. The growth restriction was not due to high concentrations of components in *L. pneumophila* spent medium or culture medium because equivalent samples lacking honey bacteria had no impact on pathogen growth, with the exception of *Shigella flexneri*, but for which the presence of honey bacteria significantly increased the ZDR (Fig. 7). The activity of the honey bacteria-produced molecules toward many but not all pathogens tested indicated that the effects were not simply due to the production of toxic compounds such as volatile organic and inorganic molecules. When examining Gram-positive pathogens, we observed ZDRs for two genera of bacteria, *Enterococcus* and *Streptococcus* (Fig. 7). These molecules were more potent toward *Streptococcus*, as AHB2 and AHB11 supernatants were able to inhibit the growth of this bacterium (Fig. S6). However, unlike for Gram-negative bacteria, growth restriction of *Enterococcus* and *Streptococcus* occurred even in the absence of AHB2 and AHB11 exposure to *L. pneumophila* (Fig. S6). These results suggested that these honey bacteria produce additional molecules independent of a *L. pneumophila* inducer that are active against a subset of Gram-positive bacteria. Collectively, our results demonstrated that while other pathogens may not induce production of an antimicrobial molecule similar to *L. pneumophila*, these molecules have broad activity against multiple, clinically relevant human pathogens, and the activity of the *L. pneumophila*-induced molecules shows specificity toward Gram-negative bacteria.

## DISCUSSION

Through a systematic evaluation of a series of bacteria isolated from raw honey, we have demonstrated their potent antibacterial activity toward *L. pneumophila* (Fig. 1). Further characterization of two of these isolates indicates that *L. pneumophila* products released into the extracellular environment instigate these bacteria to secrete antimicrobial molecules (Fig. 2) that have bactericidal activity against *L. pneumophila* (Fig. 3). Moreover, production of the antimicrobial molecules by the honey bacteria was not specific to *L. pneumophila* but a general response to multiple *Legionella* spp. and broadly active against multiple members of the *Legionella* genus (Fig. 5). In contrast, many human pathogens from diverse genera did not induce antimicrobial molecule production by the honey bacteria (Fig. 6). However, several were susceptible to the activities of the molecules (Fig. 7), including a number of high-priority pathogens. These results define specific conditions under which dormant, uncharacterized bacteria in honey produce antimicrobial molecules in response to their exposure to *Legionella* spp., revealing an untapped resource for potentially novel, broad-spectrum antibiotics.

Honey is widely recognized for its antibacterial activity, with numerous biological properties capable of preventing growth of a variety of bacterial pathogens. Here, we extend the antibacterial properties of honey to include resident bacteria that encompass at least six genera and a variety of species (Table S2). Notably, honey bacteria outgrowth from diluted honey samples was observed on *Legionella* bacteriological medium, but not Luria-Bertani (LB) medium, demonstrating media-specific requirements to define the complete honey microbiome. Since diluted honey can support the growth of bacteria (40), resident bacteria may be active at early stages of honey production when honey is less concentrated and thus, less potent. The presence of such bacteria may serve as another contributor to the antimicrobial environment of honey, possibly as part of a symbiotic relationship with honey bees to protect the hive. If so, this would extend the antimicrobial properties of honey beyond its molecular and biophysical properties to include resident bacteria and their antimicrobial products.

The interaction of several bacteria isolated from honey with *L. pneumophila* resulted in the production of potent antimicrobial molecules. More specifically, molecules produced by honey bacteria occurred in response to *L. pneumophila* filtered culture supernatant, indicating that *L. pneumophila* releases or secretes a diffusible inducer into the environment. Such a molecule could be one that competes for nutrient resources such as iron scavenging siderophores (57), molecules that typically distinguish self from non-self in bacterial populations like quorum sensing autoinducers (58), or a molecule with antimicrobial properties. Indeed, *L. pneumophila* encodes polyketide biosynthetic genes, non-ribosomal peptide synthases (59), surfactants (60, 61), an RTX toxin (62), and a Type II secretion system that secretes more than 25 substrates, including numerous lipases and proteases (63, 64). Interestingly, of the 35 pathogenic bacteria examined herein, antimicrobial molecule production by honey bacteria was only observed in response to members of the *Legionella* genus (Fig. 5 and 6), suggesting that the inducer(s) recognized by honey bacteria is unique to *Legionella* spp. or far more abundant than in other bacteria. Thus, antimicrobial molecule production by the honey bacteria is determined by what they are exposed to. While it is, as yet, unclear if individual honey bacteria respond to the same molecule, our results demonstrate that *Legionella* spp are potent inducers of antimicrobial molecule production by resident spore-forming bacilli in honey, providing a new paradigm for the identification of novel antimicrobial molecules.

While honey bacteria were generally very responsive to members of the *Legionella* genus, there were some clear distinctions. Most notably, while AHB2 and AHB11 showed potent antimicrobial activity toward all nine *Legionella* spp. tested, MHB4, AHB3, and AHB9 lacked activity against *L. longbeachae* and *L. dumoffii* (Fig. 5). One possible explanation is that AHB2 and AHB11 respond to an inducer(s) that is common to all nine species examined, whereas MHB4, AHB3, and AHB9 respond to a different inducer(s) that is not produced by *L. longbeachae* or *L. dumoffii*. Alternatively, the differences could be due to the nature of the honey bacteria antimicrobial molecules themselves (potency, target specificity, or mode of action) and/or differences in the resistance mechanisms employed by individual *Legionella* sp. to protect themselves against such molecules. Interestingly, *L. longbeachae* and *L. dumoffii* are more commonly found in soil (65–67). Physiological adaptations to this environmental niche, possibly even in direct response to antibiotic producing organisms typically found in soil, including bacilli, may confer resistance mechanisms to *L. longbeachae* and *L. dumoffii* that have not been acquired by other *Legionella* spp.

Honey bacteria showing antimicrobial activity toward *Legionella* spp. were identified by 16S sequencing as members of *Bacillus* (MHB4, AHB2, AHB3, and AHB11), *Lysinibacillus* (AHB9), and *Staphylococcus* (AHB7) genera (Table S2). *Staphylococcus epidermidis*strains are known to secrete cytolysins that can have antibacterial activities (68–70). However, while AHB7 consistently showed broad activity toward multiple *Legionella* spp. (Fig. 5), it was the least potent. In contrast, *Bacillus* and *Lysinibacillus* honey bacteria had far greater activities. Members of these genera are known producers of a variety of antimicrobial molecules, including RiPPs, NRPs, polyketides, terpenes, and volatile organic and inorganic molecules (49). The two most potent honey bacteria isolates, AHB2 and AHB11, were further identified as *Bacillus safensis*. While *B. safensis* isolates are known to be active producers of antifungals (50–52), they are not recognized as antibacterial producers, particularly against Gram-negative bacteria, with only a recent report of a soil isolate producing a lipopeptide surfactant with antibacterial activity restricted to Gram-positive *Staphylococcus epidermidis* (71). These results expand the utility of *B. safensis* in the identification of novel antimicrobial molecules and define specific culture conditions that allow the full potential of this species to be realized.

While the response of honey bacteria to produce antimicrobial products was highly specific to the *Legionella* genus, the molecules themselves had much broader activity, particularly toward Gram-negative bacteria. For example, the molecules produced by AHB2 and AHB11 in response to *L. pneumophila* were sufficient to restrict the growth of members of nine genera of pathogenic bacteria, including *Acinetobacter*, *Citrobacter*, *Enterobacter*, *Morganella*, *Providencia*, *Salmonella*, *Shigella*, *Stenotrophomonas*, and *Yersinia* (Fig. 7). The similarity in the sets of pathogens susceptible to these molecules suggests a common target, mode of action and/or lack of resistance mechanisms. These results demonstrate the potential broad utility of the honey bacteria molecules against multiple clinically important pathogens, and the importance of *Legionella*-honey bacteria interactions to access these resources.

Collectively, our results demonstrated that *Legionella* spp. are potent inducers of antimicrobial molecules produced by spore-forming bacilli isolated from honey. Thus, the interaction between these genera of bacteria may provide access to novel broad-spectrum antibiotics. Future directions will focus on defining the *Legionella*-generated inducer(s) that triggers antimicrobial molecule production by honey bacteria and the molecular mechanisms governing this response, the identity of the antimicrobial molecules produced and their modes of action, and medicinal chemistry optimization to increase their potency against other clinically important pathogens.

## MATERIALS AND METHODS

### Bacteria culture conditions

Bacteria isolated from honey (Table S2) and all bacterial pathogens examined (Table S4) were cultured at 37°C in ACES [N-(2-acetamido)-2-aminoethanesulfonic acid] buffered yeast extract (AYE) bacteriological medium or on solid charcoal ACES buffered yeast extract agar medium (CYE) (72) containing 12-g/L agar and supplemented with 0.4-g/L iron(III) nitrate, 0.135-g/L cysteine, and 0.1-mg/mL thymidine (CYET), with the exception of *L. anisa* and *L. tucsonensis*, which were cultured at 30°C, and *Streptococcus pneumoniae*, which was cultured in Mueller-Hinton medium containing 5% lysed horse blood or on solid 5% blood agar medium (Thermo Fisher Scientific). *Escherichia coli* strain DH5α (Table S4) was cultured at 37°C in liquid LB medium or on solid LB medium containing 20 g/L agar.

### Honey samples

Honey was obtained from local honey collectors through third parties (Table S1) and stored in the dark at 4°C. Honey names were given by the collectors, often based on the local flora surrounding the honey-hunting regions. For experimentation, each honey was diluted to a 50% (vol/vol) working stock solution with sterile deionized water, mixed to homogeneity, and stored at 4°C. For disk diffusion assays with *Legionella* spp., honey stock solutions were filtered with a 0.22-µm polyvinylidene fluoride (PVDF) filter. For antimicrobial property inactivation experiments, 50% stock solutions of honey were treated as follows immediately prior to use. To neutralize pH, 5 M potassium hydroxide was added to a pH of ~7 based on analysis using litmus strip tests. Reactive oxygen species were inactivated by treating honey with 1-mg/mL catalase (equivalent to 2,000–5,000 U/mL) (Sigma) at room temperature for 2 h. Proteins were inactivated by treating honey with 50-μg/mL proteinase K (Promega or Sigma) at 37°C for 30 min.

### Isolation of honey bacteria

Initial disk diffusion assays with *L. pneumophila* with 50% unfiltered honey resulted in the outgrowth of 29 different microorganisms (Table S2) based on colony morphology and pigmentation. Individual microorganisms were colony purified on CYET medium and stored in culture medium containing 20% glycerol at −80°C.

### 16S sequencing of honey bacteria

Genomic DNA was isolated from individual honey bacteria using a DNeasy Blood and Tissue kit (QIAGEN) following the manufacturer’s instructions for bacteria with the following modifications: bacteria were resuspended in 100 μL of lysis buffer [20 mM Tris-HCl, pH 8, 2 mM EDTA, 1.2% Triton-X100] containing 20-mg/mL lysozyme and incubated at 37°C for 3 h. Samples were subsequently treated with proteinase K in QIAGEN AL buffer at 56°C for 1–2 h, until bacteria were completely lysed and samples became viscous. 16S sequences were amplified by polymerase chain reaction using the 16S universal primers QUGP_F5: CCTACGGGAGGCAGCAG and QUGP_R2: GACGGGCGGTGTGTAC (73) and were sequenced. Sequence homology with BLAST (74) was used to define genus and, when possible, species, annotating the highest percent identity hit(s), which ranged from 98% to 100% (Table S2). For AHB2 and AHB11, species was further defined based on 23S, *rpoB*, *gyrB* and *recA* sequence conservation after genome sequencing (described below).

### Genome sequencing

Genome sequencing and assembly were performed at the Microbial Genome Sequencing Center (Pittsburgh, PA). Illumina and Nanopore-generated sequence read quality and adapter trimming were performed with bcl2fastq (75). Assembly was performed with Unicycler (76), and assembly statistics were assessed with QUAST (77). The genomes were annotated by National Center for Biotechnology Information using Prokaryotic Genome Annotation Pipeline (78) and deposited in GenBank with accession numbers CP097373 for AHB2 (47) and CP097374 and CP097375 for AHB11 (48).

### Disk diffusion assays

Bacteria grown to stationary phase in liquid medium were diluted in fresh medium to 10^4^ bacteria/μL, and 10^6^ bacteria were plated on solid LB medium (*E. coli*), Mueller-Hinton medium containing 5% (vol/vol) lysed horse blood (*Streptococcus pneumoniae*) or CYET medium (all other bacteria) in 10-cm petri dishes, or bacteria were diluted to 5 × 10^3^ bacterial/μL and 2.25 × 10^6^ bacteria were plated on CYET in 15-cm petri dishes to generate a lawn of bacteria. Honey diluted 50% (vol/vol) with sterile deionized water, filter sterilized 50% (vol/vol) honey, planktonic honey bacteria, or the filtered supernatant of honey bacteria cultures (sample preparation is described below and illustrated in Fig. S5) was then added to sterile 6-mm filter disks placed on the bacterial lawn. Plates were incubated at 37°C for 18–24 h for all bacteria except *Legionella* spp., which were incubated for 3 days, the time required to generate a lawn of bacteria. The ZDB outgrowth, ZDI, or ZDR for each filter disk was measured at perpendicular angles and averaged. Samples that did not promote, restrict, or inhibit bacterial growth were reported as 6 mm, the diameter of the filter disk.

Samples were prepared as follows (Fig. S5). For analyzing honey antimicrobial properties, 20 µL of deionized water (untreated) or honey (50% [vol/vol] honey, neutral pH honey, catalase-treated honey, or proteinase K-treated honey) was added to the filter disk. For analyzing the effects of individual honey bacteria, sporulating bacteria were grown to stationary phase, and 50–100 bacteria in 20-μL aliquots were plated on filter disks. For non-sporulating *Staphylococcus* bacteria (AHB7 and MHB1), 200 bacteria in 20-μL aliquots were plated on filter disks. For filtered culture supernatants, 20 μL of bacteria suspension (as described above) was used to inoculate 2.5 mL of AYE medium. Bacteria were cultured for 16–20 h at 37°C, then culture supernatants were isolated by pelleting bacteria at 13,000 rpm (13,226 *× g*) for 4 min, and harvested supernatants were passaged through a 0.22-µm PVDF filter. Concentrated, filtered supernatants were generated by concentrating filtered culture supernatants 10-fold in a Sorvall Speedvac on low heat for 3.0–3.5 h. For induction of honey bacteria antimicrobial molecule production by *L. pneumophila* culture supernatant, *L. pneumophila* was cultured to mid-log phase (*A*_600_ = 2.0). Bacteria were pelleted by centrifugation at 13,000 rpm (13,226 × *g*) for 1 min, and harvested culture supernatants were filter sterilized by passage through a 0.22-µm PES filter. The resulting *L. pneumophila* filtered culture supernatant was combined at varying ratios in a total of 1.5 mL with fresh AYE medium. Bacteria suspension (20 μL, as described above) was added and then cultured for 16–20 h at 37°C. Concentrated, filtered culture supernatants of honey bacteria exposed to *L. pneumophila* culture supernatant were then generated as outlined above. For fractionation experiments, filtered culture supernatants of honey bacteria exposed to *L. pneumophila* were sequentially passaged through centrifugal filters of 30-, 10-, and 3-kDa molecular weight cutoffs (Millipore) by centrifuging at 13,000 rpm (13,226 × *g*) at 25°C, simultaneously concentrating samples 10-fold. For the <3-kDa fraction, 10-fold concentration was performed in a Speedvac as described above. For proteolysis and thermostability experiments, concentrated, filtered culture supernatants of honey bacteria exposed to *L. pneumophila* were incubated in the presence or absence of 16-U/μL proteinase K (Sigma) at 37°C for 45 min, or incubated at 95°C for 45 min, and then used in disk diffusion assays as described above. For quality control disk diffusion assays, CLSI-recommended quality control comparator *E. coli* ATCC25922 and *Staphylococcus aureus* ATCC25923 strains were plated on Mueller-Hinton agar medium, and disk diffusion assays were performed using 10-μg gentamycin disk content, following CLSI-defined standards (79).

### Time-kill assays

*L. pneumophila* were harvested from solid medium into fresh AYE, and 5 × 10^5^ bacteria were combined with 10 μL of concentrated, filtered culture supernatants of honey bacteria exposed to *L. pneumophila* culture supernatant (generated as described above) in a total of 200 μL of fresh AYE medium. Cultures were incubated at 37°C with aeration. At regular intervals, 10-μL samples were diluted and plated on solid bacteriological medium, and the number of bacteria, based on colony forming units was enumerated. *L. pneumophila* numbers in the presence of honey bacteria supernatant were compared to control concentrated, filtered culture supernatants lacking honey bacteria and in fresh AYE alone.

## SUPPLEMENTAL MATERIAL

Figure S1 msphere.00120-24-s0001.pdf Sources of honey.

Figure S2 msphere.00120-24-s0002.pdf *L. pneumophila* is largely resilient to the antimicrobial properties of honey.

Figure S3 msphere.00120-24-s0003.pdf Outgrowth of dormant bacteria in raw honey.

Figure S4 msphere.00120-24-s0004.pdf Bacteria isolated from raw honey.

Figure S5 msphere.00120-24-s0005.pdf Method to examine antimicrobial molecule production by honey bacteria in response to *L. pneumophila* and its impact on the replication of *Legionella* spp. and other pathogens.

Figure S6 msphere.00120-24-s0006.pdf Honey bacteria produce antimicrobial molecules independent of exposure to *Legionella* that have activity against Gram-positive bacterial pathogens.

Table S1 msphere.00120-24-s0007.xlsx Honey variants.

Table S2 msphere.00120-24-s0008.xlsx Honey bacteria.

Table S3 msphere.00120-24-s0009.xlsx AntiSMASH analysis.

Table S4 msphere.00120-24-s0010.xlsx Strains.

ASM does not own the copyrights to Supplemental Material that may be linked to, or accessed through, an article. The authors have granted ASM a non-exclusive, world-wide license to publish the Supplemental Material files. Please contact the corresponding author directly for reuse.
