# Supplementary material for: Novel induction of broad-spectrum antibiotics by the human pathogen Legionella
Source: mSphere. 2024 Jun 18;9(7):e00120-24. doi: 10.1128/msphere.00120-24 (PMC11288058; doi:10.1128/msphere.00120-24)
Supplement: Figure S1 — Sources of honey. [file msphere.00120-24-s0001.pdf]

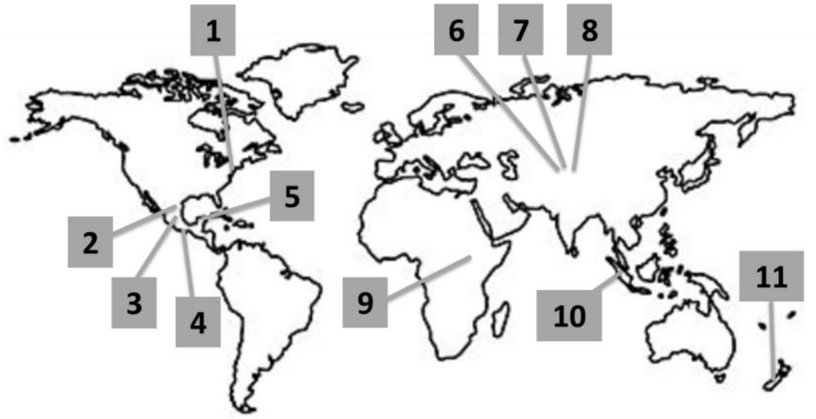

1. Clover honey, New Jersey, USA
2. Sunflower honey, Mexican Highlands
3. Texas Kidneywood honey, Guanajuato, Mexico
4. Jungle Mountain Flower honey, Oaxaca, Mexico
5. Wildflower honey, Yucatan, Mexico
6. At-Bashi honey, Eastern Kyrgyzstan
7. Issyk-Kul honey, Issyk-Kul, Kyrgyzstan
8. Sanfoin honey, Tian-Shan Mountains, Kyrgyzstan/China border
9. African honey, Kajo Keji, South Sudan
10. Tualang honey, Sumatra, Indonesia
11. Manuka honey, New Zealand

Fig. S1. Sources of raw honey. Honey was obtained from regions of North American, Mesoamerican, Central Eurasian and Southeast Asian, with different bee species, native flora and climate.
