# Supplementary material for: Novel induction of broad-spectrum antibiotics by the human pathogen Legionella
Source: mSphere. 2024 Jun 18;9(7):e00120-24. doi: 10.1128/msphere.00120-24 (PMC11288058; doi:10.1128/msphere.00120-24)
Supplement: Figure S2 — L. pneumophila is largely resilient to the antimicrobial properties of honey. [file msphere.00120-24-s0002.pdf]

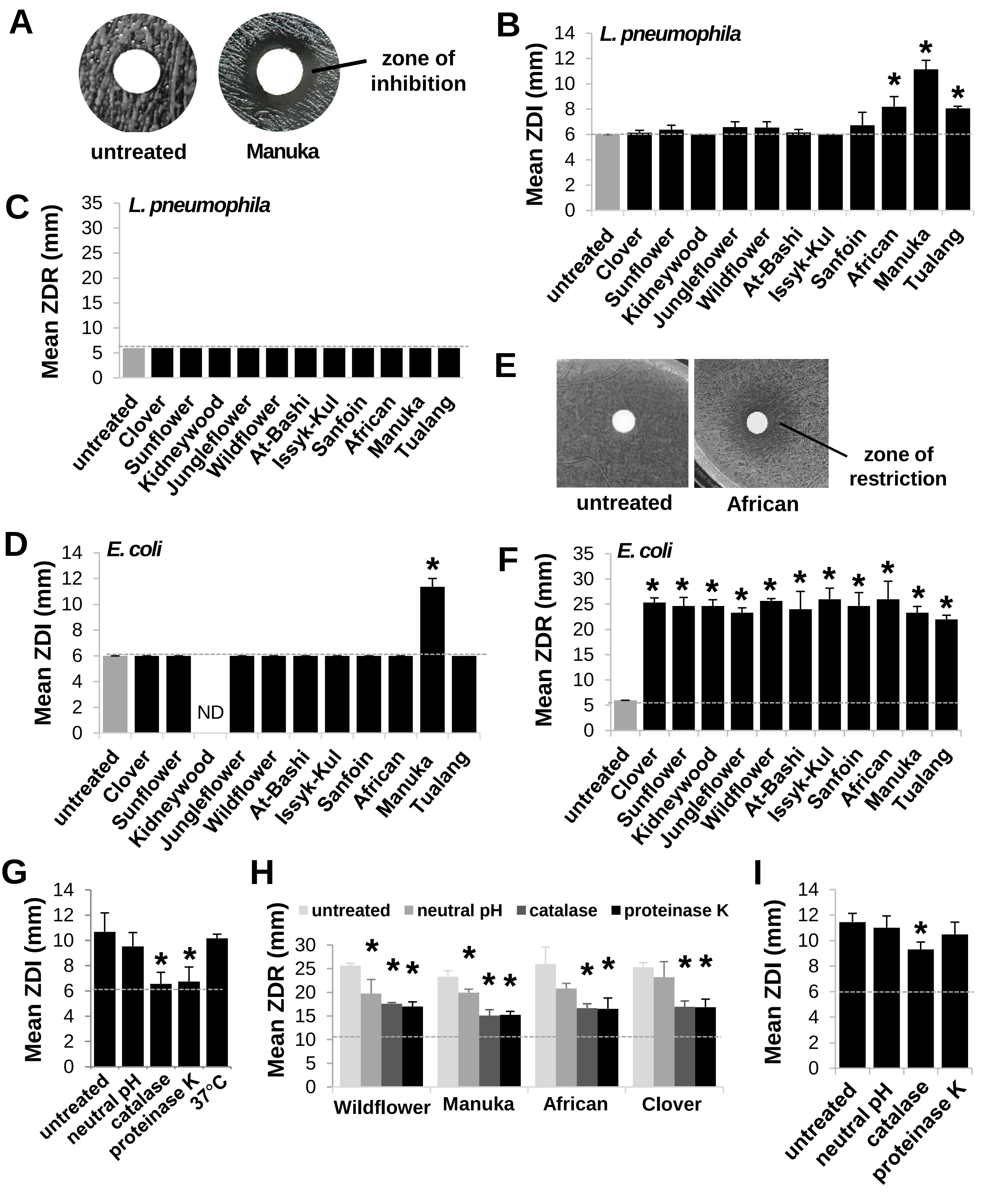

A
zone of inhibition
untreated
Manuka
B
L. pneumophila
Mean ZDI (mm)
*
*
*
untreated
Clover
Sunflower
Kidneywood
Jungleflower
Wildflower
At-Bashi
Issyk-Kul
Sanfoin
African
Manuka
Tualang
C
L. pneumophila
Mean ZDR (mm)
untreated
Clover
Sunflower
Kidneywood
Jungleflower
Wildflower
At-Bashi
Issyk-Kul
Sanfoin
African
Manuka
Tualang
E
zone of restriction
untreated
African
D
E. coli
Mean ZDI (mm)
ND
*
untreated
Clover
Sunflower
Kidneywood
Jungleflower
Wildflower
At-Bashi
Issyk-Kul
Sanfoin
African
Manuka
Tualang
F
E. coli
Mean ZDR (mm)
untreated
Clover
Sunflower
Kidneywood
Jungleflower
Wildflower
At-Bashi
Issyk-Kul
Sanfoin
African
Manuka
Tualang
G
Mean ZDI (mm)
untreated
neutral pH
catalase
proteinase K
37°C
H
untreated
neutral pH
catalase
proteinase K
Mean ZDR (mm)
Wildflower
Manuka
African
Clover
I
Mean ZDI (mm)
untreated
neutral pH
catalase
proteinase K

Fig. S2. *L. pneumophila* is largely resilient to the antimicrobial properties of honey. (A) Manuka honey inhibits *L. pneumophila* growth compared to water alone (untreated). Disc diffusion assays measuring the effects of 50% (v/v) honey on the growth of *L. pneumophila* on solid buffered charcoal yeast extract (CYE) medium. (B) Few honey variants examined inhibit the growth of *L. pneumophila*. The zone diameter of inhibition (ZDI) of *L. pneumophila* in disc diffusion assays for the indicated honey variants was quantified. Untreated samples (water control) are reported as the diameter of the filter disc alone (6 mm). (C) Honey variants do not restrict *L. pneumophila* growth. Disc diffusion assays for the indicated honey variants were performed and the zone diameter of restriction (ZDR), as defined by visibly less dense bacterial growth surrounding the filter disc (see (E) below), of *L. pneumophila* growth was quantified. (D) Manuka honey inhibits *E. coli* growth. Disc diffusion assays in which filter discs were placed on a lawn of *E. coli* grown on Luria Bertani solid medium, 50% (v/v) honey samples were individually added, and ZDIs were quantified as in (B). (E) African honey restricts *E. coli* growth compared to water alone (untreated). Disc diffusion assays measuring the effects of 50% (v/v) honey were performed as in (A) and the zone diameter of restriction (ZDR), as defined in (C), of *E. coli* was examined. (F) Multiple honey variants restrict *E. coli* growth. Disc diffusion assays for the indicated honey variants were performed and the ZDR of *E. coli* growth was quantified. (G) Growth inhibition of *E. coli* by Manuka honey depends on ROS and peptides. The major antimicrobial properties of honey include its acidity and the presence of reactive oxygen species (ROS) and antimicrobial peptides. Manuka honey was treated to neutralize each property individually and the impact on its antimicrobial activity was examined by disc diffusion assays. To do this, Manuka honey was pH neutralized (NpH), treated with catalase to inactivate ROS or proteinase K to remove peptides and then used in disc diffusion assay as in (E) and quantified as in (F). The impact of the proteinase K was not an indirect effect of heat inactivating the antimicrobial molecule during the proteinase K treatment at 37°C, as incubation of Manuka honey at 37°C alone did not reduce its activity. (H) Multiple antimicrobial properties of honey contribute to growth restriction of *E. coli*. Honey variants were treated as in (G) and then used in disc diffusion assays and the resulting ZDRs were then quantified, comparing to untreated honey. (I) Growth inhibition of *L. pneumophila* by Manuka honey partially depends on reactive oxygen species (ROS). Manuka honey was pH neutralized (NpH), treated with catalase or proteinase K as in (G) and then used in disc diffusion assay as in (A) and quantified as in (B). Neither pH neutralization or peptide removal by proteinase K treatment had any impact, whereas ROS detoxification by catalase only partially reduced Manuka honey-mediated restriction of *L. pneumophila* growth. The different impacts of these treatments on the activity of Manuka honey towards *L. pneumophila* and *E. coli* suggested the antimicrobial activity of Manuka honey against these two bacteria were due to distinct properties. (B,C,D,F-I) Data are the mean of 3 biological replicates, each consisting of 3 technical replicates. Error bars indicate ± standard deviation. An asterisk indicates a two-tailed Student's t test p value < 0.05 compared to water (B,C,D,F) or untreated honey (G,H,I), respectively.
