# Supplementary material for: Novel induction of broad-spectrum antibiotics by the human pathogen Legionella
Source: mSphere. 2024 Jun 18;9(7):e00120-24. doi: 10.1128/msphere.00120-24 (PMC11288058; doi:10.1128/msphere.00120-24)
Supplement: Figure S3 — Outgrowth of dormant bacteria in raw honey. [file msphere.00120-24-s0003.pdf]

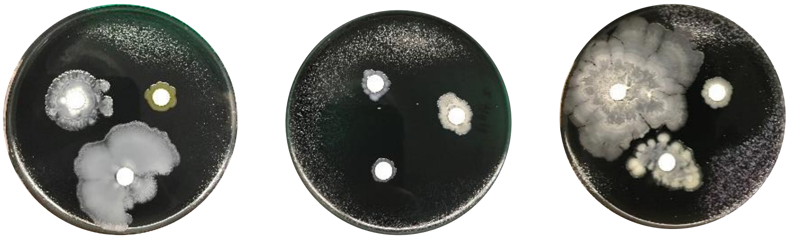

Fig. S3. Outgrowth of dormant bacteria in raw honey. Representative disc diffusion assays examining the ability of diluted, unfiltered honey to impair *L. pneumophila* replication that resulted in the outgrowth of non-*Legionella* bacteria from the filter discs.
