# Supplementary material for: Novel induction of broad-spectrum antibiotics by the human pathogen Legionella
Source: mSphere. 2024 Jun 18;9(7):e00120-24. doi: 10.1128/msphere.00120-24 (PMC11288058; doi:10.1128/msphere.00120-24)
Supplement: Figure S4 — Bacteria isolated from raw honey. [file msphere.00120-24-s0004.pdf]

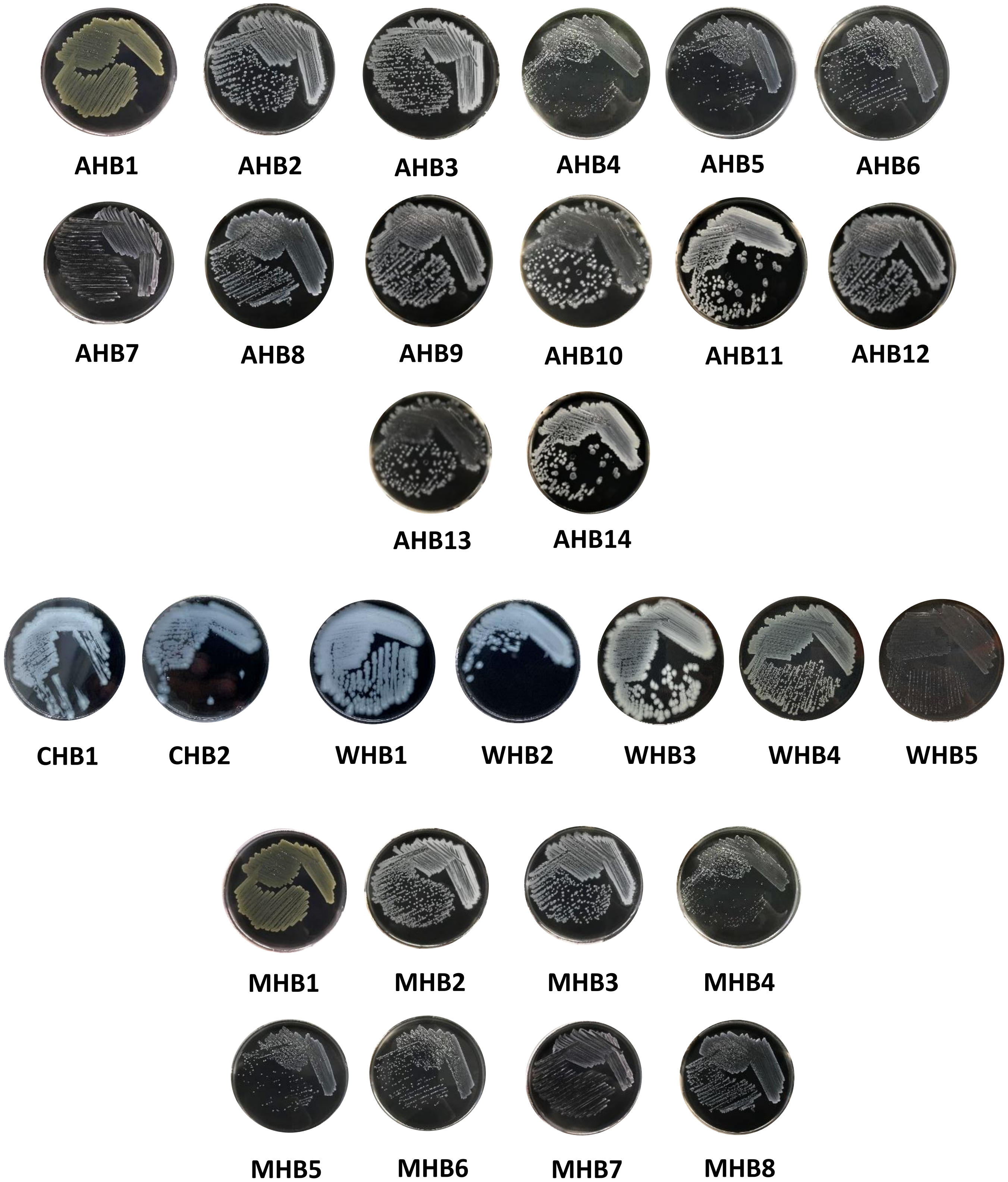


**Fig. S4. Bacteria isolated from raw honey.** Colony purified bacteria isolates from raw honey exhibit varying colony morphology, pigmentation and growth rates (based on colony size).
