# Supplementary material for: Novel induction of broad-spectrum antibiotics by the human pathogen Legionella
Source: mSphere. 2024 Jun 18;9(7):e00120-24. doi: 10.1128/msphere.00120-24 (PMC11288058; doi:10.1128/msphere.00120-24)
Supplement: Figure S5 — Method to examine antimicrobial molecule production by honey bacteria in response to L. pneumophila and its impact on the replication of Legionella spp. and other pathogens. [file msphere.00120-24-s0005.pdf]

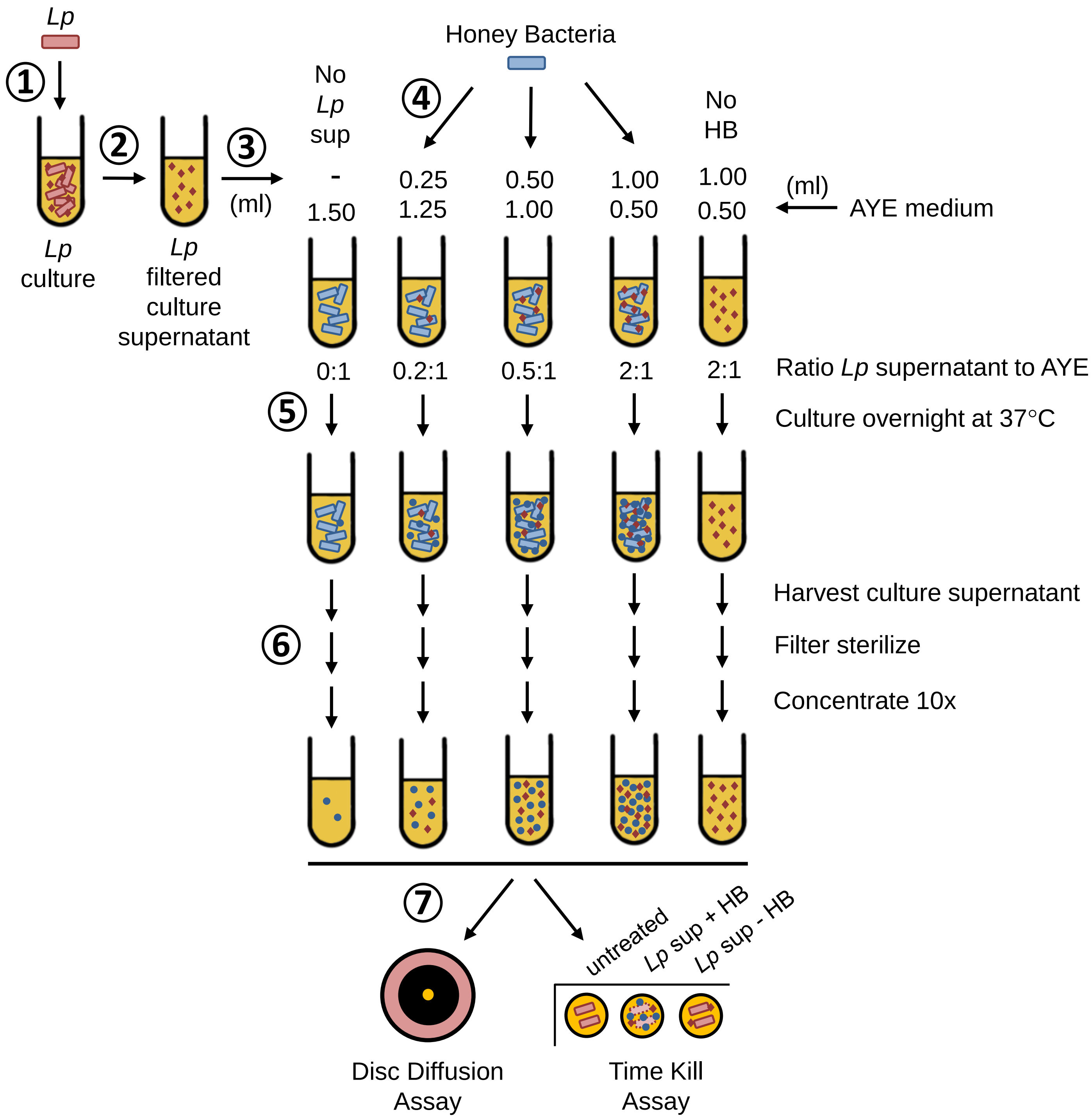

Fig. S5. Method to examine antimicrobial molecule production by honey bacteria in response to *L. pneumophila* and its impact on the replication of *Legionella* spp. and other pathogens. (1) *L. pneumophila* (*Lp*) were cultured to mid-log phase. (2) Bacteria were pelleted by centrifugation and harvested culture supernatants were filter sterilized. (3) The resulting *L. pneumophila* filtered culture supernatant (*Lp* sup) was combined at the indicated ratios with fresh AYE medium in a total of 1.5 ml. (4) Samples were then inoculated with 50-200 planktonic honey bacteria (HB) and (5) cultured overnight (16-20 hrs) at 37°C. (6) The supernatants of honey bacteria exposed to *L. pneumophila* filtered culture supernatants were harvested, filtered and concentrated 10-fold. (7) Concentrated, filtered culture supernatants of honey bacteria exposed to *L. pneumophila* filtered culture supernatant were then used in disc diffusion and time-kill assays as described in Methods, comparing to control samples lacking honey bacteria or *L. pneumophila* filtered culture supernatant.
