# Supplementary material for: Novel induction of broad-spectrum antibiotics by the human pathogen Legionella
Source: mSphere. 2024 Jun 18;9(7):e00120-24. doi: 10.1128/msphere.00120-24 (PMC11288058; doi:10.1128/msphere.00120-24)
Supplement: Figure S6 — Honey bacteria produce antimicrobial molecules independent of exposure to Legionella that have activity against Gram-positive bacterial pathogens. [file msphere.00120-24-s0006.pdf]

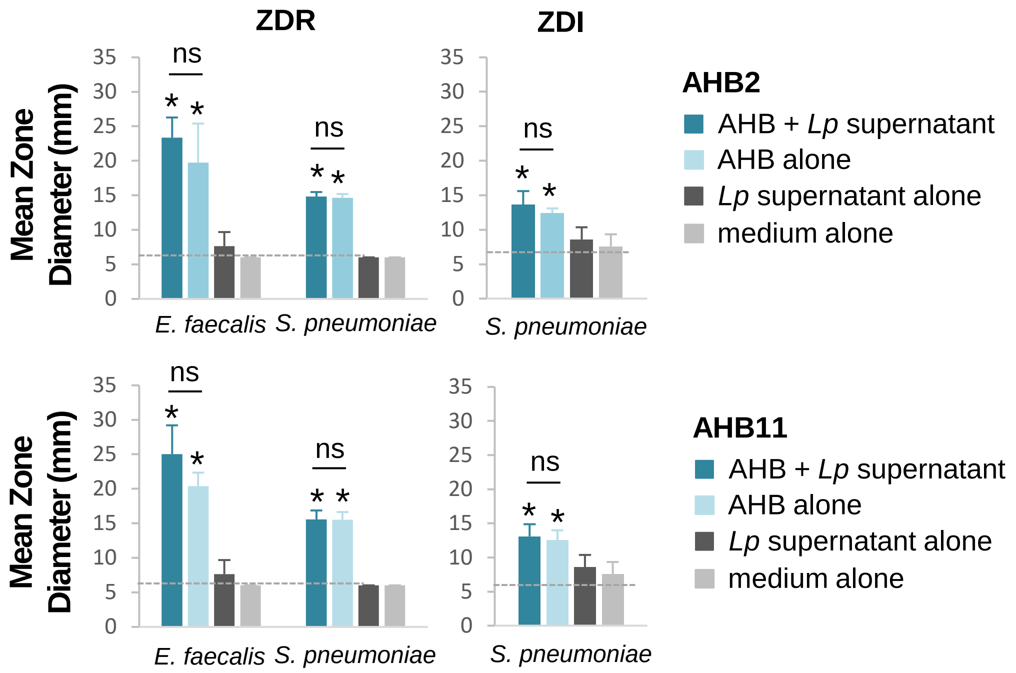


**Fig. S6. Honey bacteria produce antimicrobial molecules independent of exposure to *Legionella* that have activity against Gram-positive bacterial pathogens.** Disc diffusion assays measuring growth inhibition and/or restriction of the indicated pathogenic bacteria by concentrated, filtered culture supernatants of AHB2 (upper panels) and AHB11 (lower panels) honey bacteria in the presence or absence of pre-exposure to *L. pneumophila* (*Lp*) filtered culture supernatants. The zone diameter of pathogenic bacteria restriction (ZDR) (left panels) or diameter of pathogenic bacteria inhibition (ZDI) (right panels) was compared to the zone diameter of samples lacking *L. pneumophila* filtered culture supernatant (AHB alone), *L. pneumophila* filtered culture supernatant lacking HB (*Lp* supernatant alone) or concentrated medium alone. A dotted line indicates the diameter of the filter disc (6 mm). Data are the mean of 6-8 biological replicates. Error bars indicate ± standard deviation. An asterisk indicates a two-tailed Student's t test $p$ value < 0.05 comparing the ZDI in the presence of honey bacteria to *L. pneumophila* filtered culture supernatant lacking honey bacteria (no HB), unless otherwise indicated. ns, not significant.
